# New era for intracerebral haemorrhage management: Lessons from INTERACT3

**DOI:** 10.1002/ctm2.1419

**Published:** 2023-10-04

**Authors:** Xin Hu, Mei Fang, Chuanyuan Tao, Lu Ma, Lili Song, Chao You, Yongbo Yang

**Affiliations:** ^1^ Department of Neurosurgery West China Hospital Sichuan University Chengdu China; ^2^ The George Institute for Global Health China Beijing China; ^3^ The George Institute for Global Health, Faculty of Medicine, The University of New South Wales (UNSW) Sydney New South Wales Australia; ^4^ Department of Neurosurgery Nanjing Drum Tower Hospital The Affiliated Hospital of Nanjing University Medical School Nanjing China

1

Commentary:

Intracerebral haemorrhage (ICH) accounts for approximately 15% of all strokes throughout the world and 20%−30% of strokes in Asian countries.^1^ Although numerous efforts have been made in recent decades, there is still a lack of Level I evidence for effective ICH treatment.

Hypertension after ICH is common and is associated with haematoma expansion and poor outcome. Therefore, blood pressure lowering is theoretically promising in ICH treatment, and several well‐designed trials have been conducted. The Intensive Blood Pressure Reduction in Acute Cerebral Haemorrhage Trial 1 (INTERACT1), an international multicentre randomised trial including 404 ICH patients and comparing early intensive lowering of blood pressure (BP) (target systolic BP 140 mmHg) with standard guideline‐based management of BP (target systolic BP 180 mmHg), indicated that early intensive BP‐lowering treatment is feasible, safe and likely to reduce haematoma enlargement in ICH.^2^ Based on these results, INTERACT2 was thereafter conducted and enrolled 2839 ICH patients within 6 h from 144 hospitals in 21 countries. Although INTERACT2 did not find a significant reduction regarding death or severe disability in intensive BP lowering group, an ordinal analysis of modified Rankin scores (mRS) showed improved functional outcomes.^3^ Post hoc analyses of INTERACT2 showed that a combination of abnormal physiological parameters (systolic blood pressure, glucose and body temperature) and warfarin use was associated with worse outcomes in ICH.^4^ Guidelines for ICH management have provided recommendations regarding acute BP lowering, glucose and temperature management, as well as reversal of anticoagulation, although with different classes of recommendation and level of evidence.^5^ However, barriers still exist when implementing guidelines into clinical work, including time constraints, lack of knowledge and skills, poor motivations and adherence and so forth.^6^

The third Intensive Care Bundle with Blood Pressure Reduction in Acute Cerebral Haemorrhage Trial (INTERACT3) was an international, multicentre, pragmatic, blinded endpoint, stepped wedge cluster randomised trial conducted at hospitals mainly in low‐ and middle‐income countries. INTERACT3 aimed to investigate the effectiveness of a care bundle of active early physiological control (intensive BP lowering, glycemic control and temperature management) and reversal of anticoagulation after acute ICH. Eligible hospital sites, stratified by country and anticipated recruitment number of ICH patients during the trial period, were randomly assigned into three sequences with four periods. The intervention targets were supposed to be achieved within 1 h and maintained for 7 days.

After more than 4 years of unremitting efforts, 7036 patients from 121 hospitals in 10 countries were enrolled, with 3221 in the care bundle group and 3815 in the usual care group. The ratio of lost to follow‐up is 9.8% (374/3815) in the usual care group and 6.9% (222/3221) in the care bundle group. The baseline characteristics of the enrolled patients were well‐balanced between the two groups. As the primary outcome, patients in the care bundle group had favourable shifts in mRS scores at 6 months, compared with those in the usual care group (odds ratio .86, 95% confidence interval [CI] .76−.97; *p* = .015). Among the secondary outcomes, the care bundle group had lower 6 months mortality (odds ratio .77; 95%CI .63−.95; *p* = ·015), but the difference became insignificant after adjustment by country and patient characteristics (adjusted odds ratio .84, 96%CI .65−1.07; *p* = .16). The hospital discharge ratio by Day 7, EQ‐­5D‐­3L utility score was significantly different, while National Institutes of Health Stroke Scale (NIHSS) by Day 7 and major disability in survivors at 6 months were similar between the two groups. The estimated number needed to treat was 35 for the care bundle to prevent one patient from death or major disability. According to the Global Burden of Disease Study 2019, the absolute number for newly diagnosed ICH incidents was 3.41 million worldwide.^7^ Therefore, the implementation of INTERACT3 care bundle might prevent about 100 000 ICH patients from death or major disability.

INTERACT3 is mainly a blood pressure lowering driven trial since there is an insignificant adjusted difference regarding the glucose and temperature control or reversal of anticoagulation, although patients in the care bundle group have a higher proportion to achieve the control target of the blood glucose and body temperature. Still, there might be a synergistic effect when implementing these four aspects of the care bundle simultaneously. Multifaceted care bundle intervention design has been conducted in several studies. In a Scotland population with ischemic stroke, achieving a care bundle with evidence‐based components could reduce 30‐day and 6‐month mortality.^8^ A single­centre study in the United Kingdom compared functional outcomes before, during and after implementation of an care bundle including anticoagulation reversal, intensive blood pressure lowering, neurosurgery and access to critical care and reported that this care bundle could significantly decrease 30‐day mortality after ICH.

Due to the large sample size, patients in INTERACT3 have a broad range of baseline characteristics, which could help the generalisation of the main findings. Particularly, when compared to INTERACT2, patients enrolled in INTERACT3 has worse Glasgow Coma Scale (GCS) (12 [9−14] vs. 14 [12–15]), larger haematoma volume (15.0 mL [7.8−30.0] vs. 11 [6–20]) and more surgical intervention (26.7% vs.5.5%). Further detailed analysis focusing on the role of surgery in ICH treatment is worthy of anticipation. As an implementation study, INTERACT3 only included hospitals with no or inconsistent relevant ICH‐specific management protocols. When transferring into the intervention period, the hospitals were trained to implement the guideline‐based care bundle protocol. Consequently, INTERACT3 helped the enrolled hospitals to overcome the obstacles and increase adherence to the guideline recommendations, which contribute to the improvement of the stroke care system.

After all, INTERACT3 has brought new hope with Level I evidence for the ICH management and will promote a more active attitude instead of palliative care towards the ICH patients.
